# Rice Consumption and Urinary Arsenic Concentrations in U.S. Children

**DOI:** 10.1289/ehp.1205014

**Published:** 2012-09-24

**Authors:** Matthew A. Davis, Todd A. Mackenzie, Kathryn L. Cottingham, Diane Gilbert-Diamond, Tracy Punshon, Margaret R. Karagas

**Affiliations:** 1 Institute for Quantitative Biomedical Sciences, Geisel School of Medicine at Dartmouth, Hanover, New Hampshire, USA; 2 Section of Biostatistics and Epidemiology, Department of Community and Family Medicine, Geisel School of Medicine at Dartmouth, Hanover, New Hampshire, USA; 3 Department of Biological Sciences, Dartmouth College, Hanover, New Hampshire, USA

**Keywords:** arsenic, biomonitoring, children, dietary, exposure, NHANES

## Abstract

Background: In adult populations, emerging evidence indicates that humans are exposed to arsenic by ingestion of contaminated foods such as rice, grains, and juice; yet little is known about arsenic exposure among children.

Objectives: Our goal was to determine whether rice consumption contributes to arsenic exposure in U.S. children.

Methods: We used data from the nationally representative National Health and Nutrition Examination Survey (NHANES) to examine the relationship between rice consumption (measured in 0.25 cups of cooked rice per day) over a 24-hr period and subsequent urinary arsenic concentration among the 2,323 children (6–17 years of age) who participated in NHANES from 2003 to 2008. We examined total urinary arsenic (excluding arsenobetaine and arsenocholine) and dimethylarsinic acid (DMA) concentrations overall and by age group: 6–11 years and 12–17 years.

Results: The median [interquartile range (IQR)] total urinary arsenic concentration among children who reported consuming rice was 8.9 μg/L (IQR: 5.3–15.6) compared with 5.5 μg/L (IQR: 3.1–8.4) among those who did not consume rice. After adjusting for potentially confounding factors, and restricting the study to participants who did not consume seafood in the preceding 24 hr, total urinary arsenic concentration increased 14.2% (95% confidence interval: 11.3, 17.1%) with each 0.25 cup increase in cooked rice consumption.

Conclusions: Our study suggests that rice consumption is a potential source of arsenic exposure in U.S. children.

Arsenic is a ubiquitous metalloid found as organic and inorganic forms in nature. Although the toxicity of inorganic arsenic is well established, arsenobetaine (from fish) is considered essentially nontoxic because it passes through the body unmetabolized (Cullen and Reimer 1989; Edmonds and Francesconi 1993; Ma and Le 1998; Navas-Acien et al. 2011); other organic forms such as dimethylarsenate, arsenolipids, and arsenosugars have uncertain toxicity. Emerging evidence indicates the potential for adverse health effects from inorganic arsenic exposure at the relatively low exposure levels common to populations worldwide, including an increased risk of cancer, cardiovascular and respiratory conditions, and diabetes mellitus (including gestational diabetes) (Amaral et al. 2011; Ettinger et al., 2009; European Food Safety Authority 2009; Karagas et al. 2001, 2004; Leonardi et al. 2012; Navas-Acien et al. 2011; Sohel et al. 2009). Additionally, studies of highly exposed populations have related childhood inorganic arsenic exposure to onset of cancers and lung disease later in life (Liaw et al. 2008; Smith et al. 2006; Yorifuji et al. 2011). The developing fetus and infants may be particularly susceptible to the adverse effects of inorganic arsenic (Hall et al. 2009; Vahter 2008). More specifically, fetal exposure to inorganic arsenic has been associated with low birth weight, increased risk of infection, and higher infant mortality (Rahman et al. 2009, 2010, 2011) in more highly exposed populations. In these populations, arsenic exposure during childhood has been associated with neurobehavioral effects in cross-sectional and prospective studies (Hamadani et al. 2011; Tsai et al. 2003; von Ehrenstein et al. 2007; Wasserman et al. 2007, 2011). Little is known about the possible long-term effects of persistent low-level arsenic exposure in children.

Contaminated drinking water is a well-recognized source of inorganic arsenic (Bhattacharya et al. 2002); however, diet is the primary exposure route for people with limited exposure via drinking water (European Food Safety Authority 2009). To date, dietary exposure to inorganic arsenic in children has generally been estimated from dietary patterns and measured arsenic concentrations in food (Bastias et al. 2010; European Food Safety Authority 2009; Martorell et al. 2011; Meacher et al. 2002b; Xue et al. 2010; Yost et al. 2004). In particular, it has been estimated that children < 3 years of age have the greatest exposures to inorganic arsenic primarily due to dietary sources such as rice consumption (European Food Safety Authority 2009; World Health Organization 2011).

Rice, grains, fruits, and juices are considered the primary food sources of arsenic exposure (Meacher et al. 2002a; Xue et al. 2010; Yost et al. 2004). Both inorganic and organic forms of arsenic accumulate in rice (Mitani et al. 2009) through the silicon transport system (Ma et al. 2008) because arsenous acid (the predominant form of arsenic in flooded rice paddies) is indistinguishable from silicic acid to the rice plant. Rice cultivars show wide variation in their ability to accumulate arsenic (3- to 37-fold) (Norton et al. 2012), and the proportion of inorganic arsenic in the grain also differs according to variety (Batista et al. 2011; Williams et al. 2005, 2007). Rice grown in the United States has been shown to contain higher amounts of total arsenic and a lower proportion of inorganic arsenic [and higher organic arsenic in the form of dimethylarsinic acid (DMA)] than rice from other countries (Meharg et al. 2009; Williams et al. 2005).

Although the United States population generally consumes less rice than those of other countries, consumption has increased (Batres-Marquez et al. 2009), and processed rice products such as flours and syrups are widely used. Among the roughly one-quarter of Americans who report rice consumption, the average amount of rice consumed is approximately 1 cup of cooked rice per day (Batres-Marquez et al. 2009). Daily rice consumption varies by level of education and race/ethnicity, with the greatest rice consumption among groups other than non-Hispanic whites, particularly those of Asian descent (Batres-Marquez et al. 2009). In addition, products such as rice cereal are often the first solid foods introduced at infancy (Jackson et al. 2012).

Ingested inorganic arsenic is excreted via the kidneys within a few days of ingestion as inorganic arsenic and methylated metabolites such as monomethylarsonic acid (MMA) and DMA. Considering that methylated species also can be present in food, excreted methylated forms could represent ingestion of these moieties as well as metabolism of ingested inorganic arsenic (Molin et al. 2011; European Food Safety Authority 2009). Urinary concentration of arsenic is regarded as a valid measure of recent exposure, especially compared with methods that rely on models of exposure from dietary information (Orloff et al. 2009). Rice consumption has been related to urinary arsenic concentrations in adults (Agusa et al. 2009; Cascio et al. 2011; Cleland et al. 2009; Gilbert-Diamond et al. 2011; He and Zheng 2010; Samal et al. 2011). However, to our knowledge, the relationship between rice consumption and urinary arsenic concentrations in children has not been evaluated directly. Therefore, we examined rice as a source of arsenic exposure in U.S. children by using data on rice consumption over the 24-hr period preceding measurement of urinary arsenic concentration in the National Health and Nutrition Examination Survey (NHANES).

## Methods

We analyzed data from the NHANES survey years 2003–2008. The NHANES is a nationally representative multistage random survey of the noninstitutionalized U.S. population that is conducted by the U.S. National Center for Health Statistics. Information is gathered on health status and health behaviors through in-person interviews and detailed information is collected on diet. NHANES participants also undergo a clinical examination that includes laboratory measures such as blood and urine analyses. For this study, we used data from the NHANES demographics, in-person dietary questionnaire, physical examination, and laboratory and health questionnaire files. Because our study used publicly available and de-identified data, it was determined to be exempt from institutional board review by Dartmouth College’s Committee for the Protection of Human Subjects.

*Study population.* We analyzed data from all children (< 18 years of age) who participated in the NHANES survey from 2003 through 2008. During this period, 13,208 children participated in the NHANES, and the response rate for the entire survey was 76%. For each NHANES survey, samples from approximately one-third of the participants were randomly selected for urinary arsenic measurements. From 2003 through 2008, 2,477 children (6–17 years of age) had urinary arsenic concentrations measured in the NHANES. Of these, we excluded 154 children because of incomplete dietary information from the 24-hr recall; this yielded a final sample of 2,323 children for our study.

*Urinary arsenic assessment.* For NHANES, urine was collected from participants in arsenic-free containers and shipped on dry ice to the Environmental Health Sciences Laboratory at the National Center for Environmental Health (NCEH; Atlanta, GA) (Caldwell et al. 2009). At NCEH, urine samples were stored frozen (≤ –70°C) and analyzed within 3 weeks of collection following standardized protocols (Aposhian and Aposhian 2006; NCHS 2004). Total urinary arsenic concentrations were measured using inductively coupled plasma dynamic reaction cell–mass spectrometry on an ELAN DRC II ICPMS or Perkin-Elmer ELAN 6100 DRC plus (PerkinElmer SCIEX, Concord, ON, Canada); arsenic species and metabolites (arsenous acid, arsenic acid, MMA, DMA, arsenobetaine, and arsenocholine) were measured using high performance liquid chromatography (HPLC).

Method detection limits and interassay coefficients of variation (CV) varied among analytes and surveys. For total arsenic, the detection limit was 0.6 μg/L for the 2003–2004 survey and 0.7 μg/L for the 2005–2006 and 2007–2008 surveys. From 2003 to 2008, the detection limit was 1.7 μg/L for DMA, 0.9 μg/L for MMA, 1.2 µg/L for arsenous acid, 1.0 µg/L for arsenic acid, 0.6 µg/L for arsenocholine, and 0.4 μg/L for arsenobetaine. CV across NHANES lots varied from 3.0% to 6.1% for mean total arsenic concentrations, from 3.3% to 6.6% for DMA, and from 5.3% to 7.3% for arsenobetaine.

We focused our analyses on total urinary arsenic and urinary DMA concentrations becasue these were detected in most subjects. Urinary measurements of total arsenic and DMA, samples with levels below the detection limit (0.6% *n* = 13 and 1.0% *n* = 240, respectively) were assigned the value of the detection limit divided by the square root of 2 (Caldwell et al. 2009; Jones et al. 2011; Navas-Acien et al. 2008; Steinmaus et al. 2009). Due to uncertain or negligible health impacts of arsenobetaine and arsenocholine concentrations, we subtracted these components from the total urinary arsenic concentrations. For arsenobetaine, 48% of samples (*n* = 1,109) fell below the corresponding detection limit and were assigned the detection limit divided by the square root of 2. However, because only 23 participants (1.0%) had arsenocholine measures above the detection limit, we assigned a value of 0 to all measures that fell below the detection limit (Steinmaus et al. 2009). Thus, our definition of total arsenic included arsenous acid, arsenic acid, MMA, and DMA, consistent with previous studies (e.g., Gilbert-Diamond et al. 2011). Arsenous acid, arsenic acid, and MMA were not considered separately due to the low levels of detection (only 6.9%, 7.8%, and 40.9% of our study had values above the detection limit, respectively).

*24-hr rice consumption.* The in-person dietary questionnaire of NHANES collects detailed information on the study participant’s diet for the 24-hr period preceding the clinical and laboratory examinations (including urinary measurements) and for some measures (such as seafood consumption) up to a 30-day recall period. The NHANES 24-hr recall period is a validated assessment of dietary consumption (Moshfegh et al. 2008). At the examination, NHANES participants were asked to recall everything they ate and drank in the prior 24 hr, and NHANES staff coded these data and recorded information on the serving size. For children < 12 years of age, the dietary component was conducted with the assistance of a proxy (i.e., a parent or other caregiver), and for children 12–17 years of age the survey was administered without the assistance of a proxy.

We used U.S. Department of Agriculture (USDA) food codes to identify rice consumed during the in-person 24-hr recall period and to classify children as “rice eaters” versus “non-rice eaters.” As in previous studies, all food data from the 24-hr dietary recall period were matched to the Food Commodity Intake Database (FCID) (USDA 2010) to quantify exposure to rice (Batres-Marquez et al. 2009). The FCID provides conversion data to estimate the total content of food commodities such as rice, tomatoes, beans, and the like in each item with a USDA food code. We estimated the total amount of dry grams of rice consumed by each participant by multiplying the quantity of each food consumed during the 24-hr recall period by the FCID estimate of dry rice content (grams of rice per 100 g of food) for that specific food, and then summing across all foods consumed during the 24-hr recall period. To classify those children who consumed rice (rice eaters) versus those who did not (non-rice eaters), we operationally defined a rice eater as someone who consumed at least 0.25 cup of cooked rice (equivalent to 14.1 g white rice dry weight) in the 24-hr recall period (Batres-Marquez et al. 2009).

*Other data.* We also collected data on sociodemographics (age, sex, race/ethnicity, educational status, family income), body mass index (BMI; kilograms per meter squared), exposure to cigarette smoke, drinking-water source, and seafood consumption (obtained from both the 24-hr recall period and 30-day food recall questions). We estimated the percentages of the population that were normal weight, overweight, and obese by converting measured BMI to percentiles based on age and sex (< 85th percentile, normal; 85th to < 95th percentile, overweight; and ≥ 95th percentile, obese) (Centers for Disease Control and Prevention 2010). We anticipated that race/ethnicity would be related to rice consumption and therefore classified race/ethnicity as non-Hispanic white, non-Hispanic black, Mexican American, and other, multiple races. Due to the small number of individuals, the “other Hispanics” NHANES category was combined with the “other/multiple” race/ethnicity.

Because cigarette smoke is a potential source of arsenic exposure (Chen et al. 2004), we used serum cotinine to estimate passive or active exposure to cigarette smoke. The NHANES measures serum cotinine using an isotope-dilution HPLC/atmospheric pressure chemical ionization mass spectrometry method. For values below the detection limit of 0.015 ng/mL, a value of the detection limit divided by the square root of 2 was assigned (Jones et al. 2011; Navas-Acien et al. 2008).

We used urinary creatinine to account for urinary dilution (Barr et al. 2005). In the 2003–2004 and 2005–2006 NHANES surveys, urinary creatinine was measured on a Beckman Synchron CX3 (Beckman Coulter Inc., Brea, CA) using a Jaffe reaction, and in the 2007–2008 panel it was measured on a Roche/Hitachi Modular P (Roche Diagnostics Corp, Indianapolis, IN) using an enzymatic method. Therefore, we adjusted 2003–2004 and 2005–2006 urinary creatinine measurements to 2007–2008 equivalents (Gebel 2002; NCHS 2009).

In the United States, arsenic exposure through drinking water is found primarily in private unregulated water systems (Karagas et al. 2000; Nuckols et al. 2011). Although we were unable to obtain measurements of arsenic in drinking water for NHANES participants, we used their self-reported drinking-water source to estimate potential exposure as either public (using a community water source) or private (defined as either a well, spring, or cistern water source) water.

To exclude the possibility that seafood contributed to forms of arsenic exposure other than arsenobetaine or arsenocholine, such as DMA, we used the USDA food codes that correspond with fish, shellfish, mollusks, and/or crustaceans to identify children who consumed any seafood during the 24-hr recall period in our primary analyses [see Supplemental Material, Table S1 (http://dx.doi.org/10.1289/ehp.1205014)] (Navas-Acien et al. 2011). Furthermore, because seafood consumption may affect urinary arsenic concentration for up to 3 days, we also performed a secondary analysis in which we restricted our sample to children who reported no seafood consumption in the 30 days before urinary arsenic measurement (Molin et al. 2011) (see Supplemental Material, Table S2).

*Statistical analyses.* The NHANES uses a stratified sampling methodology that makes it possible to derive national estimates from survey participants’ data. To account for this sampling, we used complex survey design methods in Stata version 12.0 (StataCorp., College Station, TX) for all analyses. These methods account for a respondent’s probability of selection and for the NHANES sampling methodology by calculating weighting factors for each respondent that account for sampling strata, primary sampling units, and person weight variables (NCHS 2005). For all analyses we set the *p*-value for statistical significance to 0.05 (2-sided).

Because metabolic processes may vary according to a child’s age (Hall et al. 2009) and NHANES dietary data were collected differently according to age (i.e., with and without a parent or caregiver), we stratified our sample into age groups of 6–11 years and 12–17 years. Analyses were performed on all ages as well as according to these two age categories.

We log_10_-transformed total urinary arsenic (the original NHANES total arsenic measure minus arsenobetaine and arsenocholine) and urinary DMA concentrations. This transformation produced a linear association with rice consumption for total urinary arsenic (lack-of-fit *p*-value = 0.45; Draper and Smith 1981), and improved the homoskedasticity and normality of model residuals, for both total urinary arsenic and urinary DMA. For this lack-of-fit test, the null hypothesis is that there is no bias—that bias error and pure error are approximately the same; the null hypothesis thus is rejected when an *F*-statistic comparing bias error to pure error exceeds a critical value. The exponentiated model coefficients represent the relative (percent) change in the dependent variable from its mean value at the reference level of exposure (Vittinghoff et al. 2005).

Urinary creatinine can be a strong predictor of arsenic methylation efficiency; thus, we included it as an independent variable in our multiple regression models (Barr et al. 2005). However, analyses with and without creatinine yielded similar results. We adjusted for potential confounding using three different models. Our baseline adjustment model (model 1) included age (continuous), sex (boy/girl), race/ethnicity (non-Hispanic white/non-Hispanic black/Mexican American/other, multiple races), and urinary creatinine concentration (continuous). Additionally, we fit a model that further adjusted for BMI (as a continuous variable) and serum cotinine concentration (continuous) (model 2). The final model additionally adjusted for water source (public/private) and was restricted to only those children who reported no seafood consumption during the 24-hr recall period (model 3). As a secondary analysis, we repeated model 3 restricting the model to children who reported no seafood consumption in the 30-day food recall questions [see Supplemental Material, Table S2 (http://dx.doi.org/10.1289/ehp.1205014].

We used rice consumption as a predictor variable in two ways. First, we treated rice consumption as a dichotomous variable, evaluating selected population characteristics and urinary arsenic variables (total arsenic and DMA urinary concentrations) according to whether the study participant consumed ≥ 0.25 cup of cooked rice during the 24-hr recall period. To compare the characteristics of study participants we used a chi-square test for categorical variables. We then explored the potential dose–response relationship between 0.25 cup cooked rice consumed during the 24-hr recall period and log_10_-transformed total arsenic and DMA using multiple linear regression as described.

## Results

*Study participants’ characteristics according to rice eater status.* Approximately 20% (471 of 2,323 study participants) of children in our sample reported consuming at least 0.25 cup of cooked rice in the 24 hr before urinary arsenic measurement. Among children who consumed rice, total cups of cooked rice consumed per day varied from 0.25 to 3.9 cups, with a mean of 0.8 cups. Characteristics that differed according to rice consumption status included race/ethnicity and seafood consumption status (Table 1). Children who consumed rice were less likely to be non-Hispanic white (44.0% vs. 63.7%) and more likely to be classified as “other, multiple races” (23.5% vs. 9.8%) (overall *p*-value < 0.01). Rice eaters were nearly twice as likely as non-rice eaters to report having consumed at least one form of seafood during the 24-hr recall period (14.0% vs. 7.1%, *p*-value < 0.01). Overall, 66.5% of rice eaters reported having consumed seafood in the 30-day food recall questions vs. 57.6% among non-rice eaters (*p*-value = 0.03) [see Supplemental Material, Table S3 (http://dx.doi.org/10.1289/ehp.1205014]. Cotinine level also differed according to rice eater status. Among non-rice eaters 44.8% of children had serum cotinine levels ≥ 10 ng/mL vs. 36.7% among rice eaters (overall *p*-value = 0.07).

**Table 1:** Characteristics of study participants (*n* = 2,323) according to rice consumption status [% (SE)].

Non-rice eater	Rice eater^a^
No. of study participants (sample)	1,852	471
Sociodemographic characteristics
Age category (years)
6–11	46.3 (1.5)	52.1 (3.0)	0.08
12–17	53.7 (1.5)	47.9 (3.0)
Sex
Boy	49.3 (1.7)	54.2 (3.4)	0.18
Girl	50.7 (1.7)	45.8 (3.4)
Race/ethnicity
Non-Hispanic white	63.7 (2.7)	44.0 (4.1)	< 0.01
Non-Hispanic black	14.3 (1.6)	17.0 (1.9)
Mexican American	12.1 (1.5)	15.5 (2.1)
Other, multiple races	9.8 (1.5)	23.5 (3.7)
Education
Attending school	98.2 (0.4)	98.5 (0.6)	0.70
Not attending school	1.8 (0.4)	1.5 (0.6)
Annual family income (US$)
< 20,000	15.5 (1.0)	15.6 (2.1)	0.96
≥ 20,000	84.5 (1.0)	84.4 (2.1)
BMI percentilec
< 85th percentile (normal)	62.7 (1.7)	63.3 (3.0)	0.96
85th to < 95th percentile (overweight)	15.8 (1.2)	15.1 (2.2)
≥ 95th percentile (obese)	21.5 (1.3)	21.7 (2.6)
Serum cotinine (ng/mL)
< 0.015	18.3 (1.6)	23.8 (3.3)	0.07
0.015 to < 10.0	36.9 (2.2)	39.4 (2.9)
≥ 10.0	44.8 (2.4)	36.7 (3.0)
Food and drinking water
Water source
Public	81.8 (2.3)	86.1 (3.4)	0.13
Private	18.2 (2.3)	13.0 (3.4)
Seafood consumptiond
Yes	7.1 (1.0)	14.0 (2.2)	< 0.01
No	92.9 (1.0)	86.0 (2.2)
aStudy participants who reported consuming at least 0.25 cup cooked rice (equivalent to 14.1 g white rice dry weight) during the 24-hr recall period. bp-Values are for difference between non-rice eaters and rice eaters; chi-square test used in comparisons of proportions. cBMI percentile based on 2000 Centers for Disease Control and Prevention (2010) growth charts. dIncludes any fish or shellfish consumed during the 24-hr recall period before urinary arsenic measurement.						

*Urinary arsenic concentration according to rice eater status.* Total urinary arsenic (excluding arsenobetaine and arsenocholine) and urinary DMA concentrations were higher among children who reported consuming ≥ 0.25 cup rice during the 24 hr preceding urinary arsenic measurement compared with those who did not, overall, and stratified by age and by those reporting seafood consumption (Table 2). The median (interquartile range) of total urinary arsenic concentration among children who reported consuming rice was 8.9 μg/L [interquartile range (IQR): 5.3–15.6] compared with 5.5 μg/L (IQR: 3.1–8.4) among those who did not consume rice. Urinary DMA among rice eaters was nearly twice that of non-rice eaters [median, 6.0 μg/L (IQR: 3.7–10.0) compared with 3.6 μg/L (IQR: 2.1–5.1), respectively].

**Table 2:** Median (IQR) urinary arsenic concentration according to rice consumption status.

Non-rice eater	Rice eater^a^
All study participants
Total arsenic (μg/L)b	5.5 (3.1–8.4)	8.9 (5.3–15.6)
DMA (μg/L)c	3.6 (2.1–5.1)	6.0 (3.7–10.0)
Age category
6–11 years
Total arsenic (μg/L)b	5.3 (2.9–8.1)	8.6 (4.9–15.1)
DMA (μg/L)c	3.6 (2.1–5.1)	6.0 (3.9–10.0)
12–17 years
Total arsenic (μg/L)b	5.6 (3.2–8.7)	9.9 (5.9–16.5)
DMA (μg/L)c	3.5 (2.1–5.1)	6.0 (3.6–10.0)
Seafood consumptiond
Non-seafood eater
Total arsenic (μg/L)b	5.3 (3.1–7.9)	8.6 (5.1–14.7)
DMA (μg/L)c	3.4 (2.0–5.0)	5.6 (3.5–9.6)
Seafood eater
Total arsenic (μg/L)b	9.6 (4.3–18.3)	17.3 (7.4–29.3)
DMA (μg/L)c	5.1 (3.1–8.2)	9.8 (5.6–18.6)
aStudy participants who reported consuming at least 0.25 cup cooked rice (equivalent to 14.1 g white rice dry weight) during the 24-hr recall period. bExcludes arsenobetaine and arsenocholine; 13 study participants with total arsenic concentrations below the limit of detection (LOD) were assigned values equal to LOD/√–2. c240 study participants with concentrations below the LOD for DMA were assigned values equal to LOD/√–2. dIncludes any fish or shellfish consumed during the 24-hr recall period before urinary arsenic measurement.				

*Urinary arsenic concentration and amount of estimated rice consumption.* Log_10_-transformed urinary total arsenic and DMA increased with rice consumption (Table 3). In the models adjusted for participant characteristics, serum cotinine, and urinary creatinine concentration (model 1), each 0.25 cup of rice consumption was associated with a 14.3% [95% confidence interval (CI): 10.2, 18.5%] and 13.5% (95% CI: 10.3, 16.9%) increase in urinary total arsenic and DMA concentration, respectively. Estimates from models further adjusted for BMI, cotinine, and water source and restricted to children who did not report seafood consumption were similar (Table 3).

**Table 3:** Estimated percent change (95% CI) in urinary arsenic concentration per 0.25 cup of daily rice consumption by age category.

Model 1^a^	Model 2^b^	Model 3^c^
All study participants
Total arsenicd	14.3 (10.2, 18.5)	13.5 (9.6, 17.5)	14.2 (11.3, 17.1)
DMAe	13.5 (10.3, 16.9)	12.9 (9.9, 16.0)	13.4 (10.5, 16.4)
Age category
6–11 years
Total arsenicd	22.0 (15.7, 28.7)	19.9 (14.7, 25.4)	16.1 (11.6, 20.7)
DMAe	19.9 (14.7, 25.3)	18.1 (14.0, 22.3)	14.7 (10.5, 19.0)
12–17 years
Total arsenicd	10.7 (6.6, 14.9)	10.5 (6.4, 14.9)	12.8 (9.2, 16.5)
DMAe	10.7 (7.5, 14.0)	10.7 (7.4, 14.1)	12.5 (8.7, 16.4)
All models include daily rice consumption as per 0.25 cup cooked rice (continuous) and predict log10-transformed urinary arsenic concentration (all parameter estimates are exponentiated). aModel 1 adjusted for age (continuous), sex (boy/girl), race/ethnicity (white/black/Mexican-American/other), and urine creatinine level (continuous). bModel 2 further adjusted for BMI (continuous) and serum cotinine level (continuous). cModel 3 further adjusted for water source (public/private) and restricted to study participants who reported no seafood consumption during the 24-hr recall period. dTotal arsenic excludes arsenobetaine and arsenocholine; 13 study participants with total arsenic concentrations below the limit of detection (LOD) were assigned values equal to LOD/√–2. e240 study participants with concentrations below the LOD for DMA were assigned values equal to LOD/√–2.						

Estimates for the effects of rice consumption on total urinary arsenic concentration differed by age category (ages 6–11 years vs. 12–17 years) (Table 3). Each 0.25 cup of rice consumption was associated with a 22.0% (95% CI: 15.7, 28.7%) increase in total urinary arsenic among children in the younger age category compared with a 10.7% (95% CI: 6.6, 14.9%) increase among those in the older age category in model 1. Differences in the estimated effect of rice consumption between the age categories persisted in our other models, albeit attenuated in model 3. In our secondary analyses, restricted to only children who reported no seafood consumption in the preceding 30 days, we obtained similar results [see Supplemental Material, Table S2 (http://dx.doi.org/10.1289/ehp.1205014). Estimates for urinary DMA in relation to rice consumption also differed by age category (Table 3). Rice consumption was associated with a 19.9% (95% CI: 14.7, 25.3%) increase in urinary DMA among children in the younger age category compared with a 10.7% (95% CI: 7.5, 14.0%) increase among those in the older age category. However, this age group difference was less apparent in model 3 (14.7%; 95% CI: 10.5, 19.0% vs. 12.5%; 95% CI: 8.7, 16.4% respectively).

*Urinary arsenic concentration and other factors.* In the multiple regression models excluding those who reported seafood in the prior 24 hr, rice consumption was the strongest independent predictor of total urinary arsenic concentration (Table 4). Both age and urinary creatinine also were statistically significant predictors of total urinary arsenic concentration. Each 1-year increase in age was associated with a 5.2% decrease (95% CI: –6.9, –3.6%) in total urinary arsenic concentration. Total urinary arsenic concentration varied by race/ethnicity, but the association was statistically significant only for Mexican Americans (13.0% higher than non-Hispanic whites; 95% CI: 1.0, 26.5%). Each increase in nanograms per milliliter of serum cotinine level was associated with a 0.7% increase (95% CI: 0.6, 0.8%) in total urinary arsenic concentration.

**Table 4:** Estimated percent change (95% CI) in total urinary arsenic concentration according to covariates from univariate and multiple linear regression models.

Covariate	Univariate			Multiple linear regression^a^		
	Estimated percent change (95% CI)	^p^-Value	Estimated percent change (95% CI)	^p^-Value
Rice consumption (0.25 cup cooked rice)	15.6 (12.2, 19.1)	< 0.001	14.2 (11.3, 17.1)	< 0.001
Age (years)	0.4 (–1.4, 2.2)	0.66	–5.2 (–6.9, –3.6)	< 0.001
Sex
Boy	0.0 (reference)	0.0 (reference)
Girl	–17.0 (–24.7, –8.5)	< 0.001	–8.5 (–17.2, 1.2)	0.08
Race/ethnicity
Non-Hispanic white	0.0 (reference)	0.0 (reference)
Non-Hispanic black	29.2 (12.4, 48.5)	< 0.01	–1.3 (–12.2, 11.0)	0.82
Mexican American	18.8 (3.1, 36.8)	0.02	13.0 (1.0, 26.5)	0.03
Other, multiple races	35.9 (8.8, 69.8)	0.01	4.9 (–12.1, 25.3)	0.59
BMI (kg/m2)	0.6 (–0.3, 1.6)	0.17	0.0 (–1.2, 1.1)	0.95
Serum cotinine (ng/mL)	0.0 (–0.1, 0.1)	0.76	–0.1 (–0.2, 0.1)	0.22
Urinary creatinine (mg/L)	0.6 (0.5, 0.7)	< 0.001	0.7 (0.6, 0.8)	< 0.001
Water source
Public	0.0 (reference)	0.0 (reference)
Private	7.3 (–15.4, 36.0)	0.56	16.4 (–10.7, 51.7)	0.26
All analyses restricted to study participants who reported no seafood consumption during the 24-hr recall period and predict total urinary arsenic concentration. Total arsenic excludes arsenobetaine and arsenocholine; 13 study participants with total arsenic concentrations below the limit of detection (LOD) were assigned values equal to LOD/√–2. aAdjusted for all other covariates in table.								

## Discussion

In this nationally representative study of U.S. children, we found that urinary arsenic concentrations—a biomarker of recent arsenic exposure—were associated with reported rice consumption in the 24 hr before urine collection. These findings are consistent with other recent studies that have examined rice as a source of dietary exposure to arsenic in adult populations (Agusa et al. 2009; Cascio et al. 2011; Cleland et al. 2009; He and Zheng 2010; Samal et al. 2011). In the United States, studies of specific populations have reported more than three times the national average of 6.0 μg/L in high-rice-consuming individuals (i.e., Korean Americans) (Cleland et al. 2009). Also, findings from this study of children corroborate those of our previous study of rice consumption and urinary arsenic concentrations among pregnant women in the United States (Gilbert-Diamond et al. 2011).

At present, the health effects of low-level arsenic exposure are uncertain, especially in children, and no studies have specifically evaluated the potential health effects of arsenic in rice to our knowledge. Previous studies have associated childhood exposure to high levels of inorganic arsenic, primarily from drinking water, with numerous adverse health effects, including neurobehavioral effects such as reduced vocabulary and object assembly skills (von Ehrenstein et al. 2007), attention and memory (Tsai et al. 2003), and intelligence (Wasserman et al. 2004). However, it is currently unknown whether low levels of arsenic exposure, or exposure from arsenic intake via rice specifically, have similar effects.

Rice consumption varies among individuals and among subgroups of the population. Higher rice consumption among racial/ethnic minorities such as those of Asian descent and populations with lower income and less education has been reported previously (Batres-Marquez et al. 2009), consistent with patterns observed in our study of children who participated in NHANES. Rice and rice products may constitute an appreciable portion of the diet in young children (World Health Organization 2011) and among people on wheat-free diets [e.g., celiac disease patients (Ludvigsson and Green 2011)]. As we reported recently, certain toddler formulas containing brown rice syrup had relatively high concentrations of arsenic (Jackson et al. 2012). Thus, measurement of biomarkers in children may help us determine common sources of arsenic exposure, such as via rice and rice products, and whether these sources pose a health risk.

A number of limitations of our study must be acknowledged. First, seafood (including fish, shellfish, mollusks, and/or crustaceans) is a well-recognized source of arsenic, particularly the organic forms such as arsenobetaine, arsenosugars, arsenolipids, and DMA (Navas-Acien et al. 2011). To minimize the effect of arsenic ingestion from seafood, we restricted a subset of our analyses (model 3) to children who reported no seafood consumption of any kind during the 24-hr recall period, and performed secondary analyses restricted to children who did not consume seafood in the previous 30 days [see Supplemental Material, Table S2 (http://dx.doi.org/10.1289/ehp.1205014]. However, our results were robust to this more conservative exclusion of seafood eaters for up to 30 days before urinary measurement.

Second, as mentioned, our estimates of rice consumption do not directly translate into estimates of arsenic consumed because of the large variation in arsenic concentrations in rice (Batista et al. 2011; Williams et al. 2005, 2007). Collecting information on the type of rice consumed (e.g., brown, white, or other varieties) would allow for better estimation of the association between rice intake and arsenic exposure in the future. Such information further could improve our understanding of the extent to which the DMA in urine results from the metabolism of inorganic arsenic versus the excretion of DMA from rice itself; these two pathways are indistinguishable in our study.

Third, we likely underestimated rice consumption. The addition of a diverse range of rice products to processed foods makes it difficult to accurately assess total rice consumption in the United States. Rice bran, rice flour, rice starch, and rice syrup are often added to products, including breakfast cereals aimed at children, cereal bars, and gluten-free products, and these products may contain arsenic (Jackson et al. 2012). Therefore, our estimates of the relationship between urinary arsenic concentrations and rice consumption are likely to be conservative because of errors in our estimate of rice consumption itself, which could have biased our parameter estimates towards the null.

Despite these limitations, our findings suggest that rice is a potential source of arsenic exposure in U.S. children and highlight the need to better understand the health consequences of common levels of arsenic exposure early in life.

## Supplemental Material

(135 KB) PDF
