# Supplementary material for: Rice Consumption and Urinary Arsenic Concentrations in U.S. Children
Source: Environ Health Perspect. 2012 Sep 24;120(10):1418–24. doi: 10.1289/ehp.1205014 (PMC3491944; doi:10.1289/ehp.1205014)
Supplement: (135 KB) PDF [file ehp.1205014.s001.pdf]

Supplemental Material

Rice Consumption and Urinary Arsenic Concentrations in US Children

Matthew A. Davis; Todd A. Mackenzie; Kathryn L. Cottingham; Diane Gilbert-Diamond; Tracy Punshon; and Margaret R. Karagas

Contents

Supplemental Material, Table S1: The United States Department of Agriculture Food Codes used in this Study to Identify Seafood Consumption[a] in the 24-hour Recall Period. 3
Supplemental Material, Table S2: The Estimated Percent Change in Urinary Arsenic Concentration per $\frac{1}{4}$ Cup of Daily Rice Consumption for Study Participants who Reported No Seafood Consumption in the 24-hour Recall Period Compared to the 30-day Food Recall Questions Prior to Urinary Arsenic Measurement. 4
Supplemental Material, Table S3: The Percentage of Study Participants who Reported Seafood Consumption in the 24-hour Recall Period and the 30-day Food Recall Questions According to Rice Eater Status. 5

. **Supplemental Material, Table S1: The United States Department of Agriculture Food Codes used in this Study to Identify Seafood Consumption[a] in the 24-hour Recall Period.**

USDA Food Code	Description
Any Seafood (yes/no)	
26100000 to 26999999	Fish and shellfish including mollusks
27150000 to 27159999	Seafood creams, ceviche, stews
27250000 to 27259999	Seafood cakes, soups, rices
27350000 to 27359999	Seafood soups, pots, salads
27450000 to 27459999	Seafood salads and vegetables
27550000 to 27559999	Seafood sandwiches
28150000 to 28159999	Seafood frozen meal
28350000 to 28359999	Seafood soups
32105020	Omelet with fish
41811200, 41811850	Fish, scallops & meatless
58010154, 58117410	Taco, tostada with fish, codfish fritter
58134310, 58145120	Pasta with seafood
58149210, 58409000	Somen salad with fish, noodle soup with fish
58151100 to 58151199	Sushi
75127500, 75232000, 75232050, 75513010, 75647000	Seaweed

Abbreviations: USDA, United States Department of Agriculture

[a] Navas-Acien A, Francesconi KA, Silbergeld EK, Guallar E. 2011. Seafood intake and urine concentrations of total arsenic, dimethylarsinate and arsenobetaine in the US population. Environ Res 111(1):110-118.

- **Supplemental Material, Table S2: The Estimated Percent Change in Urinary Arsenic Concentration per $\frac{1}{4}$ Cup of Daily Rice Consumption for Study Participants who Reported No Seafood Consumption in the 24-hour Recall Period Compared to the 30-day Food Recall Questions Prior to Urinary Arsenic Measurement.**

	Estimated Percent Change (95% CI)	
	No Seafood in 24-hour	No Seafood in 30-day[c]
All Study Participants		
Total Arsenic[a]	14.2 (11.3, 17.1)	17.6 (10.3, 25.3)
Dimethylarsinic Acid[b]	13.4 (10.5, 16.4)	14.5 (8.2, 21.2)
Age Category		
6 to 11 years		
Total Arsenic[a]	16.1 (11.6, 20.7)	17.1 (8.9, 26.0)
Dimethylarsinic Acid[b]	14.7 (10.5, 19.0)	14.1 (5.5, 23.3)
12 to 17 years		
Total Arsenic[a]	12.8 (9.2, 16.5)	16.6 (9.0, 24.7)
Dimethylarsinic Acid[b]	12.5 (8.7, 16.4)	13.5 (5.8, 21.8)

Abbreviations: CI, confidence interval
All models include daily rice consumption as per $\frac{1}{4}$ cup cooked rice (continuous) and predict $\log_{10}$-transformed urinary arsenic concentration (all parameter estimates are exponentiated). All models adjusted for age (continuous), sex (boy/girl), race/ethnicity (White/Black/Mexican-American/Other), urine creatinine level (continuous), body mass index (continuous), serum cotinine level (continuous), and water source (public/private).
[a] Total arsenic excludes arsenobetaine and arsenocholine. 13 study participants with total arsenic concentrations below the limit of detection (LOD) were assigned values equal to $\frac{LOD}{\sqrt{2}}$
[b] 240 study participants with concentrations below the LOD for dimethylarsinic acid were assigned values equal to $\frac{LOD}{\sqrt{2}}$
[c] 737 study participants had incomplete data for the NHANES items that inquired about "any seafood" or "any shellfish" consumed in the past 30 days.

- **Supplemental Material, Table S3: The Percentage of Study Participants who Reported Seafood Consumption in the 24-hour Recall Period and the 30-day Food Recall Questions According to Rice Eater Status.**

	Percent (SE)		
	Non-rice Eater	Rice Eater	p-value[a]
Seafood Consumption in 24-hour			
Yes	7.1 (1.0)	14.0 (2.2)	<0.01
No	92.9 (1.0)	86.0 (2.2)	
Seafood Consumption in 30-day[b]			
Yes	57.6 (2.4)	66.5 (3.8)	0.03
No	42.4 (2.4)	33.5 (3.8)	

Abbreviations: SE, standard error
Seafood consumption during the 24-hour recall period was identified by using the United States Department of Agriculture Food Codes in Supplemental Material, Table S1, whereas seafood consumed within 30-day period was determined by National Health and Nutrition Examination Survey items that inquired about "any seafood" or "any shellfish" consumed in the past 30 days.
[a] p-values are for the difference between Non-rice Eaters and Rice Eaters, χ^2 used in comparison of proportions.
[b] 737 study participants had incomplete data for the NHANES items that inquired about "any seafood" or "any shellfish" consumed in the past 30 days.
